# Malaria Prevention, Mefloquine Neurotoxicity, Neuropsychiatric Illness, and Risk-Benefit Analysis in the Australian Defence Force

**DOI:** 10.1155/2015/287651

**Published:** 2015-12-17

**Authors:** Stuart McCarthy

**Affiliations:** Headquarters 2nd Division, Australian Army, Randwick Barracks, Randwick, NSW 2031, Australia

## Abstract

The Australian Defence Force (ADF) has used mefloquine for malaria chemoprophylaxis since 1990. Mefloquine has been found to be a plausible cause of a chronic central nervous system toxicity syndrome and a confounding factor in the diagnosis of existing neuropsychiatric illnesses prevalent in the ADF such as posttraumatic stress disorder and traumatic brain injury. Overall health risks appear to have been mitigated by restricting the drug's use; however serious risks were realised when significant numbers of ADF personnel were subjected to clinical trials involving the drug. The full extent of the exposure, health impacts for affected individuals, and consequences for ADF health management including mental health are not yet known, but mefloquine may have caused or aggravated neuropsychiatric illness in large numbers of patients who were subsequently misdiagnosed and mistreated or otherwise failed to receive proper care. Findings in relation to chronic mefloquine neurotoxicity were foreseeable, but this eventuality appears not to have been considered during risk-benefit analyses. Thorough analysis by the ADF would have identified this long-term risk as well as other qualitative risk factors. Historical exposure of ADF personnel to mefloquine neurotoxicity now also necessitates ongoing risk monitoring and management in the overall context of broader health policies.

## 1. Introduction

Two of the most significant threats to the health of Australian Defence Force (ADF) personnel are vector-borne diseases such as malaria [1–3] and environmental or operational stress, which can cause a variety of psychiatric disorders [4–6]. The ADF commits extensive resources to address these risks including the areas of research, training, prevention, diagnosis, and treatment [1, 3, 5–7]. In the case of malaria, preventive medications such as doxycycline, atovaquone-proguanil, primaquine, and mefloquine play an important role in overall preventive health strategies [3]. However recent insights into mefloquine's neurotoxic properties, chronic neuropsychiatric adverse effects, and factoring in neuropsychiatric illness [8–10] make it timely to reassess the benefits of using the drug for malaria prophylaxis against the risks of causing or aggravating neuropsychiatric illness, or otherwise complicating the management of mental health, in the ADF population.

Mefloquine hydrochloride (trade name* Lariam*) is a 4-quinolinemethanol synthetic quinoline that has been used to treat chloroquine resistant* P. falciparum* malaria [11], although since its introduction into the market in the late 1980s and early 1990s it has mainly been used for malaria prophylaxis [12–16]. The drug is prescription-only and the manufacturer states that when used “in chemoprophylaxis the safety profile of mefloquine is characterised by a predominance of neuropsychiatric adverse reactions” [12]. Concerns over the frequency and severity of these neuropsychiatric reactions have been a subject of controversy since its introduction [14, 16–18]. Although there are other adverse effects, the neuropsychiatric effects remain the focus of this paper.

Mefloquine was found to be neurotoxic in 2006 [19], although uncertainties remain as to dosages, idiosyncratic effects, and precise biochemical mechanisms of action [8, 9, 19–24]. More recently, it was found that mefloquine prophylaxis can cause a chronic central nervous system (CNS) toxicity syndrome evident in a number of other quinolines historically used as antimalarials and antiparasitics [9]. This finding synthesized a body of clinical observations, pharmacoepidemiological findings, and experimental neuropharmacological evidence to describe a syndrome of symptoms linked to neuronal injury particularly in the vestibular system and brainstem, establishing mefloquine CNS toxicity as a plausible cause of acute and chronic neuropsychiatric symptoms [8, 9] previously attributed to other causes [17, 24]. Medical authorities have also found that mefloquine prophylaxis can confound the diagnosis of neuropsychiatric illnesses prevalent in the ADF including posttraumatic stress disorder (PTSD) and traumatic brain injury (TBI) [9, 25].

Mental health disorders among ADF serving personnel and veterans have been extensively studied, including risks associated with operational stress, environmental stress, and the use of nonprescription drugs [26, 27]. Mental health management has undergone significant policy reform, including the implementation of a number of risk management measures [5, 6, 28]. Policies have been introduced to manage a variety of specific neuropsychiatric illnesses including PTSD and TBI [29, 30]. Pharmaceutical risk-benefit analysis (RBA) in this context extends beyond individual decisions by clinicians and patients to include more challenging organisational and policy level analysis and interdisciplinary decision making. ADF preventive health doctrine includes guidance on risk assessment [1] and more broadly the ADF adopted the organisation-wide, systematic Australian Defence Risk Management Framework in 2003 [31, 32].

This paper critically reviews the use and risk management of mefloquine in the ADF in light of the drug's ability to cause or aggravate neuropsychiatric illness, with reference to the literature on RBA, neurotoxicology, and other relevant disciplines. The inclusive term* neuropsychiatric* is used advisedly in this paper, in relation to causal factors, drug effects, symptoms, disorders, and sequelae [33], noting the mental health focus of this edition.* Mental health* is commonly used exclusively in relation to psychiatric disorders resulting from environmental stressors. However the paper also considers disorders with neurobiological causes such as neurotoxicity or physical forces causing neuronal damage, due to their overlapping symptomology, comorbidity, and prevalence in the ADF population. Many of the relevant drug effects and symptoms are also commonly described as* neuropsychiatric* in the existing literature; hence the use of this term is inclusive of* mental health* disorders.

## 2. Methods

The review originated with the premise that the recent description in the medical-scientific literature of a previously unrecognized chronic CNS toxicity syndrome [8, 9], which can be caused by a drug in the ADF pharmaceutical inventory [3, 12], necessitates a reappraisal of the risks associated with the drug's use. Conducting an actual RBA is beyond the scope of this paper in the absence of the necessary medical records and other data; however it was determined that a comprehensive literature review would provide a useful summary of the evidentiary basis from which a reappraisal of risk by policy makers is to be initiated. The review required both a prospective and a retrospective approach to risk analysis and evidence: prospective in the sense of identifying risks associated with continued use of the drug, and new or established approaches for addressing risks arising from the exposure of significant numbers of personnel to a neurotoxic agent; retrospective in the sense of reappraising existing assumptions, policies, or practices relating to historical use of the drug that may now be invalidated by the evidence of mefloquine neurotoxicity.

Within the methodological framework described below, the general approach to identifying relevant material was to search PubMed, Scopus, and Google Scholar using search terms such as drug names, chemical names, symptoms, disorders, and author names. Previously published meta-analyses and literature reviews provided a baseline to initiate the literature search. Several experts in epidemiology, tropical medicine, pharmacology, and neurotoxicology were also consulted to assist in identifying relevant material. This was an invaluable aspect of the review given its broad, interdisciplinary scope.

The first subject of the literature search was pharmaceutical RBA, focusing on the organisational or policy level in comparison to individual clinical settings [34]. There is extensive literature on this subject, including the use and interpretation of qualitative versus quantitative evidence [35–38] in relation to study design [39–41], including for drugs with idiosyncratic adverse effects [41]. Given that neuropsychiatric effects were evident in mefloquine's safety profile early in its history [14, 16] and that concerns over veterans' exposure to neurotoxicants including medicines became prominent in the 1990s [42–44], the literature search then included the discipline of neurotoxicology. This literature describes the manifestation, symptomology, and evidentiary basis of toxic encephalopathies [45–48], neurotoxicity syndromes [49, 50], methods for neurotoxicity testing [51–53], and risk assessment [54–57]. This section of the review provided a general frame of reference within which the search and analysis of literature relating specifically to mefloquine toxicity is refined.

The two papers cited in the introduction that describe the chronic mefloquine CNS toxicity syndrome [8, 9] were then closely examined, including the material cited in those papers, to determine the analytical approach used by the authors to find that mefloquine prophylaxis is able to cause lasting toxic injury to the CNS with chronic sequelae. This established that the findings were a synthesis of historical medical-scientific evidence relating to the toxicity of quinolines, with more recent evidence drawn from three related disciplines, namely, clinical observation, pharmacoepidemiology, and experimental neuropharmacology. The search and analysis of literature for the next section of the review was therefore structured in accordance with those four lines of investigation.

The literature identified in each of those four areas was then critically analysed, beyond the literature cited in the two papers, initially by examining published clinical case reports and related investigations [58–72], systematic reviews [16, 17, 24], meta-analyses [73, 74], and experimental neuropharmacological studies [19–23] relating to the safety and tolerability of mefloquine prophylaxis, either conducted by or cited by public or military health authorities. A further search was then conducted to identify published pharmacoepidemiological studies relating to the safety and tolerability of mefloquine prophylaxis in healthy adult travellers and military personnel from developed countries [16, 75–92], including all studies conducted by the ADF [85–88], as well as longitudinal or follow-up studies relating to the original study populations [89] or those who had previously submitted adverse event reports to a national drug regulator [90]. Most of the studies include reporting of acute and subacute effects during or immediately following prophylaxis [16, 75–89, 91, 92]. However one study of chronic psychiatric effects was identified and this was limited to individuals who had submitted adverse event reports to a national drug regulator [90]. Two studies that include both treatment and prophylaxis are relevant because they provided a basis for widely cited estimates for the incidence of neuropsychiatric adverse events, including prophylaxis [16, 92]. Studies relating exclusively to treatment doses or use in specific groups such as children or pregnant women were not considered in detail. Another study was selected because it examined user acceptability, with a high proportion of mefloquine respondents citing convenient weekly dosing as the main reason for their choice [75]. Although the review initially set out to include civilian studies of long-term prophylaxis that included clinical observation of the subjects, a paucity of these in the literature led to the selection of these civilian studies merely to illustrate a variety of study methodologies. This section of the review analysed the evidentiary basis of mefloquine's adverse toxic properties as it evolved through the history of the drug's development and use, in the contemporaneous literature available to policy makers involved in RBA.

One key area of dispute that became apparent by this stage of the review was underestimation [16, 92] or systemic underreporting of adverse effects in pharmacoepidemiological studies [74, 75]. A retrospective statistical analysis of these studies was considered unwarranted as it would bring little value to the literature. However it was determined that a qualitative assessment of the various study methodologies could provide valuable insight. A number of the studies incorporated aspects of observational study [76–78, 83, 90] and reported results that could inform future study design, although in all but one case [90] these were limited to assessing acute or subacute neuropsychiatric effects during or shortly after prophylaxis. Summarising the strengths and limitations of these study methodologies became a key focus of the review.

A complete search of all Australian Repatriation Medical Authority (RMA) determinations was then conducted to identify those that list mefloquine or quinolines as causal factors in service related diseases. The RMA is an independent, statutory medical authority whose determinations are legal instruments used to assess eligibility for veterans' entitlements. The relevant legislation recognizes a disease only where it is chronic or recurrent and explicitly excludes “a temporary departure from the normal physiological state,” that is, transient, acute conditions [92, 93]. The standard of evidence used by the RMA is medical or scientific publication subjected to a peer-review process, and standard epidemiological criteria are used in their assessment of causation [92]. Although the RMA has yet to publish a determination on the mefloquine CNS toxicity syndrome, its recognition of mefloquine or quinoline exposure as causally related to other neurological and psychiatric conditions provides a useful indication of the availability of published evidence to policy makers.

The review then examined all available ADF health policies, doctrine, and major research studies relevant to malaria prevention, risk management, and mental health. There is extensive literature on the prevalence of psychiatric or mental health disorders in ADF personnel [26, 27] and veterans [27, 94], and substantial policy reforms have been made in this area [5, 6, 28, 29], although the literature search indicates that the use of prescription medications as a possible causal factor has to date been excluded from consideration. No studies on the prevalence of neurological disorders in the ADF could be identified since a study of 1991 Gulf War veterans in relation to medical and chemical exposures, which showed increased reporting of neurological symptoms; however the study does not indicate which malaria prophylaxis regimens were used [42]. The comprehensive review of ADF health policies and doctrine included those relating to preventive health [1], malaria [3], mental health, and psychiatric illness [4–6, 27, 28]. This included examining specific policies on the management of PTSD [29] and TBI [30], given recent findings that mefloquine prophylaxis can confound the diagnosis of those prevalent conditions [10, 25].

Finally, a critical analysis of this body of the literature then deduced a number of qualitative risk factors that could reasonably have been included in RBA relating to mefloquine prophylaxis in the ADF, both in general use and specifically in drug trials, with reference to contemporaneous medical-scientific literature. In the case of drug trials, further reference was made to the applicable international standard for good clinical practice, mandated under the relevant Australian legislation [95]. One limitation of this review is that actual RBA relating to mefloquine use in the ADF is not publicly available; however ADF malaria policy [3] and published papers on the drug's historical use in the organisation [2, 3, 15, 85–88] provide sufficient insight to inform this analysis in that the risk of neuropsychiatric adverse effects is cited as a reason for limiting the drug's use.

## 3. Risk-Benefit Analysis and Neurotoxicology

### 3.1. Interdisciplinary Risk-Benefit Analysis at the Policy or Organisational Level

The practice of RBA, which is defined as “examination of the potential positive and negative results of undertaking a specific therapeutic course of action” [96], is a cornerstone of medical practice including preventive medicine. In a civilian context this is typically the domain of individual judgement by a patient and/or prescriber, balancing therapeutic efficacy with safety risks to prevent or treat a single illness, relying principally on information from the manufacturer and drug regulators [34, 36, 38]. While there is often only a single benefit, there may be multiple risks even for an individual. Perceptions of risks versus benefits are also greatly influenced by context and may therefore differ from actual risks and benefits [35].

In military organisations such as the ADF, RBA is more complex because it requires a broader analysis of context and organisational factors, drawing upon the considerable resources of its health system including a capacity to conduct internal research and/or commission independent research. This necessitates an interdisciplinary approach in which expertise is drawn from all relevant disciplines, broadening the assessment in response to new evidence as necessary [31, 32]. The process is not static but requires ongoing reevaluation of the risk-benefit balance as greater knowledge of a drug's efficacy and adverse effects is obtained throughout its life cycle [35, 36]. This is emphasised in ADF preventive health doctrine, which states that “evaluation is an ongoing process [which] provides medical staff with feedback on the accuracy of hazard identification and the consecutive risk assessment” [1].

The literature on mefloquine indicates that the present policies relating to the drug's safety have been based principally on pharmacoepidemiological studies [3, 7, 8, 12–14, 16–18, 24, 73–92]. From an interdisciplinary perspective, however, early reports of neuropsychiatric reactions in 1989 [16], subsequent direct evidence of neurotoxicity in 2006 [19], and the more recent description of a chronic CNS toxicity syndrome in 2013-2014 [8, 9] would each have warranted a broadening of this approach to include the discipline of neurotoxicology [49–57]. In retrospect, incorporating the methodologies of that discipline into subsequent studies would likely have resulted in a better understanding of the drug's properties, health impacts, and risks than is currently the case.

### 3.2. Qualitative versus Quantitative Evidence and Study Design

Viewed narrowly within the discipline of pharmacoepidemiology, there is a wealth of literature on the interpretation of quantitative versus qualitative evidence in RBA by researchers, clinicians, regulators, and policy makers [35–41]. Quantitative aspects of RCTs are prominent in RBA throughout the life cycle of a drug; however there are key limitations. Although safety data can be gathered, overall safety cannot be fully determined within RCTs because a drug's safety profile involves multiple safety issues [37, 39, 40]. In the case of individual RCTs, design of the trial can limit its internal validity in that specific adverse effects can only be assessed once they have been observed [39]; then they can be ignored or disregarded if assumed to be idiosyncratic [41]. The external validity of the trial can then be further limited by the homogeneity of the trial subjects [37, 39, 40]. For these reasons, an interdisciplinary approach to pharmacoepidemiological study using both RCTs and observational studies is important in understanding a given drug's safety profile early in its use [40].

Regardless of any interdisciplinary considerations, regulators typically assess quantitative data from RCTs and post-market reporting, as well as qualitative evidence from clinical case studies and pharmacovigilance activities such as adverse event reports as a drug is used more widely [34, 37]. More extensive use of the drug over time is also important as it is exposed to a larger population, of broader heterogeneity compared to earlier trials, with a longer duration of exposure [37–40]. Long-term exposure is critical in understanding a drug's safety profile, particularly so with adverse effects such as chronic organ toxicity [37].

### 3.3. Toxic Encephalopathies and Neurotoxicity Syndromes

The term toxic encephalopathy refers to brain dysfunction caused by toxic exposure. This includes a spectrum of symptomologies ranging from subclinical deficits to overt clinical disorders. The clinical manifestations of toxic encephalopathy are related to the affected brain regions and cell types. Neurotoxic chemicals capable of damaging the CNS are quite prevalent, including heavy metals, organic solvents, and other industrial chemicals. Many of these have been found to cause relatively specific neurological syndromes including diffuse acute or chronic toxic encephalopathy, chronic solvent encephalopathy, cerebellar syndrome, parkinsonism, and vascular encephalopathy [45, 46]. There are a number of well-known iatrogenic (pharmaceutical) causes of toxic encephalopathy, for example, some cancer chemotherapeutics [97, 98] and psychotherapeutics [47]. In some cases, including higher treatment doses of mefloquine and other quinolines [99, 100], the neuropsychiatric symptoms of the iatrogenic encephalopathy are difficult to distinguish from those of the disease being treated [45, 46].

The discipline of neurotoxicology recognizes a number of fundamental principles that are relevant to this consideration of mefloquine. Firstly, compared to toxic diseases of other organs, the nervous system's limited regenerative capacity means that more sequelae persist after the removal of a neurotoxic agent. Secondly, multiple neurological syndromes may occur in response to a single neurotoxic agent, depending on the level and duration of the exposure. Thirdly, few neurotoxic agents result in pathognomonic neurological syndromes. CNS clinical disorders instead have varying presentations involving a host of nonspecific symptoms, with the symptoms of neurotoxic exposure often mimicked by various other neuropsychiatric diseases [45]. These provide a useful frame of reference for the literature relating specifically to mefloquine.

### 3.4. Neurotoxicology and Risk Assessment

The discipline of neurotoxicology became prominent in the latter part of the 20th century, as advances were made in the neurosciences and widespread health impacts of common environmental and industrial neurotoxic agents such as heavy metals, solvents, and pesticides became apparent. By the 1990s, insights into the development and application of neurobehavioral toxicology methods saw the adoption of standardised neurobehavioral test batteries, neuroimaging techniques, biochemical markers, questionnaire studies, and epidemiological studies of neurotoxic disorders [51–53]. Similarly, standardised neurotoxicity risk assessment practices have been in place since the mid-1990s [54–57].

The nature of CNS and peripheral nervous system disorders is such that the patient is commonly unaware of the relationship between his/her symptoms and possible causes and may not recognize changes in his/her behaviour until they are brought to his or her attention by family or coworkers. Nonspecific effects of neurotoxicants include headache, nausea, and dizziness. When patients among a group are exposed to neurotoxicants, the effects may vary from one to another because of differences in susceptibility and other risk factors [54]. This suggests that even pharmacoepidemiological studies that include neurobehavioural observations would not necessarily be able to make accurate causal attribution in the absence of clinical investigation of individual patients using the appropriate methods, particularly when the pathophysiology of the toxic agent in question has yet to be described in the literature, and where its symptoms mimic those of other prevalent conditions. Further, this would warrant inclusion of qualified neurotoxicologists in RBA processes as soon as there are indications that a pharmaceutical product may be linked to CNS injury.

## 4. Development, Use, and Safety of Mefloquine

Mefloquine was developed by the US military's Walter Reed Army Institute of Research (WRAIR) during the 1970s, mainly in response to the onset of chloroquine resistant* Plasmodium falciparum* malaria in Southeast Asia [8, 9, 14]. The drug's synthesis was first reported in the literature in 1971 [101]. Initially trialled on prisoners, soldiers, and subjects in developing countries [8, 14], mefloquine's ongoing use and development has been closely linked to military requirements and operations [9, 14]. After licensing and introduction into the civilian market in the late 1980s and early 1990s it became widely used for chemoprophylaxis, favoured over other efficacious drugs for the convenience of its once weekly dose [13, 16, 17], with more than 20 million people having taken the drug worldwide [3]. Notably, initial licensing occurred in the absence of phase III clinical safety and tolerability trials in a normal study population of healthy civilian volunteers [14], although various trials have been subsequently undertaken [8, 14, 73, 74, 76, 77]. During the mid- to late-1990s concerns were raised over the frequency and severity of mefloquine's acute adverse neuropsychiatric effects, including reports of hallucinations, psychosis, and suicidal behaviour, with the drug's safety attracting controversy since that time [8, 14, 17, 18]. Nonetheless the drug remained first-line malaria prophylaxis in numerous military forces for many years, including the US until 2009 [102], and to date in Canada [103] and the UK [104].

Mefloquine was introduced into the ADF antimalarial inventory in 1990 [105]. The drug has been used principally for suppressive chemoprophylaxis in personnel contraindicated for the ADF's first-line prophylactic doxycycline, initially as a second-line agent [85, 86, 106, 107] and currently as third line [3]. Approximately 5–10% of ADF personnel do not tolerate doxycycline [3]. Another quinoline drug, primaquine, is used for terminal prophylaxis to eradicate any residual liver stages of vivax malaria [3]. Current ADF malaria policy notes that mefloquine is contraindicated for personnel with preexisting psychiatric illness and prohibits specialist personnel including aircrew and divers from using the drug, citing the acute adverse neurological effects. The policy attributes concern over the drug's safety to “public perception” [3]. Documented uses of mefloquine by the ADF have occurred since 1988, including clinical trials conducted by the Army Malaria Institute (AMI) during training exercises in malarious countries [85] and deployments to United Nations peacekeeping missions in Somalia and Cambodia [86]. The largest documented populations of ADF recipients were administered the drug during AMI clinical trials in East Timor from 2000 to 2002, totalling more than 1,300 personnel [87, 88]. Although overall historical figures are not publicly available, these published figures combined with the numbers of ADF personnel deployed to malaria endemic areas since 1990, as well as the proportion of personnel who do not tolerate doxycycline, place the overall total in the thousands.

The manufacturer currently cites a randomized control trial (RCT) in which treatment-related neuropsychiatric adverse events occurred in 28.8% of the mefloquine recipients, with the affected percentages including strange or vivid dreams, 13.7%; insomnia, 13.5%; dizziness or vertigo, 8.9%; visual difficulties, 3.3%; anxiety, 3.7%; and depression, 3.5% [12]. Post-marketing data is also cited by the manufacturer to report the incidence of neuropsychiatric adverse effects. Psychiatric disorders include very common (>1/10), abnormal dreams and insomnia; common (≥1/100 to <1/10), anxiety and depression; and uncommon (≥1/1,000 to <1/100), agitation, restlessness, mood swings, panic attacks, confusional state, hallucinations, aggression, bipolar disorder, psychotic disorder including delusional disorder, depersonalisation and mania, paranoia, and suicidal ideation. Neurological disorders include common (≥1/100 to <1/10), dizziness, headache, and vertigo; uncommon (≥1/1,000 to <1/100), balance disorder, somnolence, syncope, convulsions, memory impairment, peripheral sensory neuropathy and peripheral motor neuropathy (including paraesthesia, tremor, and ataxia), encephalopathy, and vestibular disorders (long-term) including tinnitus and hearing impaired [12].

Despite evidence of quinoline CNS toxicity dating back to the 1940s [9], numerous early reports linking mefloquine use to a variety of acute psychotic events [8, 9, 58–60], and reports of toxic encephalopathy [58], direct evidence of mefloquine neurotoxicity was not established until a series of experimental studies were conducted well over a decade after the drug's introduction [19–23]. Developers appear to have assumed for considerable time that neurological effects from the 4-quinolinemethanol class were only transient [108, 109]. Direct evidence eventually published in 2006 found that mefloquine is neurotoxic, causing brain stem lesions that are “permanent in nature” in animal models at dosages equivalent to those used in malaria treatment [19]. Further studies have shown mefloquine neurotoxicity in animal neurons [20, 21] and human neuronal cell lines [22, 23]. Clinical observations following prophylaxis have also shown behavioural effects consistent with lasting cognitive impairment symptomatic of neurotoxic brainstem lesions [8, 9, 61]. A recently published review synthesized the above findings with studies of historically used quinolines to describe mefloquine neurotoxicity as “chronic sequelae of a well characterised but idiosyncratic central nervous system toxicity syndrome…associated with a risk of permanent neuronal degeneration within specific central nervous system regions including the brainstem” [9]. The same author has elsewhere described mefloquine neurotoxicity as a cause of neurotoxic vestibulopathy [110].

There is no explicit acknowledgement from the manufacturer that mefloquine can cause the neurotoxicity syndromes listed above; however product information warns that during prophylactic use “signs of unexplained acute anxiety, depression, restlessness or confusion…may be considered prodromal to a more serious event,” in which case “the drug must be discontinued” [12]. No definition of “a more serious event” is offered; however this statement has significant safety implications. Acknowledging barriers to recognition and reporting of such symptoms that are examined in Section 7.3, it is considered reasonably likely that a significant proportion of military users, among others, would continue taking the drug and experience such unspecified “serious events.” Further, such a statement may constitute tacit rather than explicit acknowledgement by the manufacturer of the drug's neurotoxicity and potential causality in chronic neuropsychiatric disorders. Exemplifying what some authors describe as “miscoding” of data including serious adverse effects by pharmaceutical companies [35, 36], the statement may be “code” for “neurotoxic.”

The research community, drug regulators, and policy makers appear to be gradually accepting the finding that mefloquine is neurotoxic. As early as 2006, for example, researchers associated with WRAIR stated that the institute “is currently investigating mefloquine analogues, seeking one with similar efficacy but reduced neuropsychiatric toxicity” [111]. In the same year, the US Army solicited private industry proposals “to define the biological mechanisms of mefloquine neurotoxicity, identify genetic and other predispositions to mefloquine neurotoxicity, and identify whether mefloquine neurotoxicity may extend to other anti-malarials as a class effect” [112]. More recently, in 2013, the US Food and Drug Administration (FDA) updated its public information for mefloquine, mandating its most serious “black box” warning, to advise in part that “neurologic side effects can occur at any time during drug use, and can last for months to years after the drug is stopped or can be permanent” [113]. There now appears to be little doubt that the drug is able to cause lasting or irreversible injury to the CNS, rather than merely transient neuropsychiatric effects, as was previously accepted [3, 9, 13, 16–18].

## 5. Mefloquine CNS Toxicity

### 5.1. CNS Toxicity of Historical Quinolines

The Australian military became directly involved in development, use, and research of synthetic quinolines, in conjunction with the US military, during the Second World War. Disruption of quinine supplies [114], coupled with a high rate of malaria casualties in the South West Pacific in 1942-43, led to the establishment of an army medical research unit which was the forerunner of the AMI [115, 116]. This unit conducted clinical experiments and trials with alternative quinolines in Northern Australia and was responsible for the first identification of human malaria drug resistance [117, 118].

The recent description of a mefloquine-induced chronic CNS toxicity syndrome [9] draws upon evidence of CNS toxicity in three quinolines historically used as antimalarials or antiparasitics, namely, pamaquine, plasmocid, and clioquinol [9]. Pamaquine is an 8-aminoquinoline that was the first drug to be synthesized with a marked activity against human malaria parasites [110]. In 1945 this drug was trialled by the Australian military as prophylaxis against New Guinea strains of* P. vivax*, finding that it did not prevent primary attacks but did prevent relapses [117, 119]. A number of pamaquine clinical trials were undertaken by the US Army Medical Department, which reported the incidence of severe toxic reactions at 1–10%, including “symptoms referable to the central nervous system, principally headache, dizziness, ‘nervousness,' psychosis, and coma” [120]. A 1949 postmortem examination of one case involving fatal overdose found significant neuronal degeneration within specific brain structures including the brainstem [9, 121]. Neurological reactions to pamaquine similar to those observed in clinical trials were also observed in animal testing involving low doses, including histopathology that revealed swelling and subtle degeneration in neurons throughout various brainstem nuclei [9, 122].

At the time of Australian military research into pamaquine, another quinoline known as quinacrine (also known as atebrine and mepacrine) had become the main malaria prophylaxis drug, protecting against* P. vivax* and* P. falciparum* [115–118], although an outbreak of the latter in one area of Northern New Guinea led to the discovery of quinacrine resistance [117]. Quinacrine was used at various dosages for both treatment and prophylaxis, with dosages altered in response to overseas findings of adverse neuropsychiatric reactions [118]. A series of case reports and studies since the mid-1930s had documented toxic psychiatric reactions including psychosis, mania, schizophrenia, depression, lassitude, and insomnia [123–126]. Some of the same studies also observed broader symptoms possibly causally related to CNS toxicity rather than peripheral causes, consistent with the pathophysiology and symptomology of the quinoline CNS syndrome described above, including anorexia and tachycardia [126]. Australian military personnel involved in medical treatment of quinacrine users also observed numerous neuropsychiatric symptoms including neuropathies and psychosis [127].

Australian military malaria research ceased soon after the war but resumed in the mid-1960s as chloroquine resistance became apparent in Southeast Asia. The AMI was established in 1973 and has been directly involved in numerous research initiatives related to the toxicity of quinolines including chloroquine, primaquine, mefloquine, and tafenoquine, in close association with WRAIR, other research institutions, and the pharmaceutical industry [15, 85–88, 128, 129].

### 5.2. Clinical Investigations

Numerous case reports linking mefloquine prophylaxis to a variety of neuropsychiatric conditions have been published since the drug was introduced into the market. These have been summarised elsewhere, particularly those relating to acute psychotic reactions [8, 9]. The present review set out to compare the body of case studies and related research linking mefloquine prophylaxis to recognized psychiatric, neurological, and other disorders, in order to assess whether the recently described mefloquine induced chronic CNS toxicity syndrome [9] may represent a distinguishable neurotoxicity syndrome consistent with the principles summarised in Section 3.3, in particular that a single toxic agent may cause multiple neurological syndromes of varying presentations, involving a variety of nonspecific symptoms, often mimicked by other neuropsychiatric diseases [45]. This was aided by a complete search of statutory determinations previously made by the RMA, which uses epidemiological criteria in their assessment of causation and peer-reviewed publication in the medical-scientific literature as their standard of evidence [93]. Additionally, the manufacturer lists a number of these same disorders as adverse effects associated with mefloquine prophylaxis, as a reflection of adverse event and clinical case reporting [12, 34].

One of the early case reports relating to mefloquine use involved toxic encephalopathy [60]. A more recent report described a case of limbic encephalopathy and central vestibulopathy, citing much of the literature reviewed here [61]. The RMA are yet to publish a determination relating to mefloquine as a cause of toxic encephalopathy, although it is interesting to note that they have previously recognized chronic solvent encephalopathy as a diagnosable disease [130]. This may provide a useful guide on any future determinations regarding mefloquine CNS toxicity. Notwithstanding any remaining disputes or uncertainties regarding mefloquine toxicity, use of synthetic quinolines has been independently determined by the RMA to be a causal factor in a variety of psychiatric, neurological, vestibular, and cardiac diseases [131–141]. These are strikingly similar to a list of conditions that the US Department of Veterans' Affairs had identified from case reports in a document advising of possible long-term health effects of mefloquine as early as 2004 [142]. Reporting of psychiatric disorders linked to mefloquine use includes cases of depression [62, 63, 131], anxiety [62, 64, 132], bipolar disorder [65, 133], and suicide or suicidal ideation [64, 66, 134]. Reporting of neurological disorders linked to mefloquine use includes cases of neuropathies [67–69, 135, 136], vertigo [62, 64, 137], and hearing loss or tinnitus [70, 139, 140]. Case reporting has also causally linked mefloquine to tachycardia [70, 72, 141]. One interesting observation about this list of disorders is that, although mefloquine is a known ototoxicant [143, 144], there is as yet no direct evidence that it can injure the peripheral nervous system; therefore a number of the recognized peripheral disorders may be more plausibly attributed to the CNS toxicity syndrome previously described [8, 9].

One specific case worthy of mention here is the report of a 1993 postmortem examination of brain tissue specimens from several US military personnel who died while serving in Somalia, where mefloquine was used for chemoprophylaxis. The examiners found mefloquine concentrations of 14 mg/kg, 8.7 mg/kg, and 11 mg/kg in the tissue samples provided [145], findings that are important to the discussion in Section 5.4 regarding the assumed dose dependence of mefloquine neuropsychiatric adverse effects.

### 5.3. Pharmacoepidemiological Studies

The understanding of any drug's properties typically improves over time as it is administered to a wider population of users and subjected to more extensive research and reporting [34, 35, 37, 39]. Many authors express concern regarding overemphasis by policy makers on quantitative data in preference to qualitative evidence epitomized by the clinical observations and experimental studies cited elsewhere throughout this review, emphasising a need for caution in RBA [36–38] particularly in cases where drug reactions are idiosyncratic or alternative efficacious therapies are available [37, 41]. Proper study design and implementation early in a drug's use is a critical aspect of gaining that understanding while remaining cognisant of safety, and there are good avenues for incorporating observational methods into the process [39, 40]. This warrants a critical analysis of quantitative estimates on the frequency of neuropsychiatric adverse effects associated with mefloquine prophylaxis, including early estimates of the incidence of adverse events and the methodologies of subsequent pharmacoepidemiological studies.

Responding to a series of early neuropsychiatric adverse event reports in 1989, the World Health Organisation (WHO) and F. Hoffmann-La Roche conducted a collaborative study to identify the characteristics of reported cases, measure the frequency of adverse events, and generate hypotheses on risk factors relating to mefloquine safety. Interim guidelines were issued including “a warning statement that persons operating machinery and those requiring fine coordination (e.g., airline pilots) should not take mefloquine prophylaxis” [16]. The 1991 report estimated a “frequency of central nervous system disorders from mefloquine [that was] crudely calculated.” Prophylaxis use figures were estimated based on sales data and a series of assumptions relating to the proportion drugs sold for treatment versus prophylaxis, the proportion of drugs sold versus actual usage, estimates of malaria treatment based on actual reported cases modified by a factor of two, and an assumption about duration of travel. The estimate of adverse events was made using a total of 140 actual reports related to mefloquine prophylaxis, modified by a factor of two in order to account for underreporting which in the jurisdictions under consideration varied from 50% to 90%. A ratio of serious versus nonserious adverse events related to prophylaxis was then estimated by reference to the actual adverse event reports and assumptions about dose dependence, even though “50 (41%) had taken a single 250 mg dose prior to the onset of symptoms” and “there was no statistical difference between the doses taken by patients with serious and non-serious adverse events.” Thus it was calculated that 1 : 10,000 prophylaxis users would experience serious neuropsychiatric adverse events [16]. An independent study published in the same year arrived at a similar figure of 1 : 13,000 but noted that “our denominator is too high, and the real incidence of side effects may be greater than that revealed in our study” [92].

The history of mefloquine's development and widespread use by the military is critical in that quantitative data from military phase III drug trials informed early estimates of adverse events in the absence of more appropriate civilian trials and has continued to influence regulatory and policy decisions [3, 16, 73, 74]. A major 1997 study conducted a meta-analysis of RCTs comparing mefloquine with other standard malaria prophylaxis drugs, which was subsequently revised, updated [73], and then incorporated into a broader analysis of common antimalarials in 2009 [74]. Ten trials were selected, involving a total of 2,750 adult participants. Five of those were field trials involving mainly male military personnel in a peacetime training setting. Withdrawals were consistently higher in four placebo controlled trials, and in five trials there was no difference in tolerability between mefloquine and the comparator drugs [73]. 516 published case reports of mefloquine adverse effects were identified, including four fatalities, mainly in tourists and business travellers. Significantly, the report makes a number of observations suggesting the limited generalisability of the trial results, noting the predominance of fit, young, male soldiers among the total number of subjects.

The report concluded that, given evidence from nonrandomized studies of its potentially harmful neuropsychiatric effects in civilian travellers, mefloquine “has adverse effects that limit its acceptability” [73]. The study was not able to determine whether the drug is well or poorly tolerated. In response to the earlier, widely cited WHO/F. Hoffmann-La Roche estimate of 1 : 10,000 users experiencing severe neuropsychiatric reactions, the report states that figure “undoubtedly underestimates the true incidence” of less severe adverse effects. Significantly, the report recommended that an international panel of experts be convened to research and resolve the question of mefloquine safety [73]. The subsequent 2009 report stated that “soldiers are a healthy and disciplined study population who, compared to non-soldiers, are likely to under-report adverse events,” resulting in “systemic under-estimation of the true frequencies” of adverse effects [74]. This observation is further informed by particular military barriers to reporting neuropsychiatric adverse drug effects including symptom recognition, stigma, and cognitive function, which are identified in Section 7.4 with reference to the Australian military literature.

This section of the review addresses the question of systemic underestimation by examining the reporting and attribution methodologies of a number of pharmacoepidemiological studies, with further reference to the above literature on neurotoxicology. Fifteen studies were examined, involving a total population of 10,664 mefloquine prophylaxis subjects during the period 1988 to 2006. These include three RCTs [76–78], six nonrandomized field trials [79–81, 85–88], and six longitudinal, cross-sectional, or retrospective studies of varying designs [75, 82–84, 90, 91]. Severe adverse events were generally defined as those requiring medical intervention, with reporting of nonsevere adverse events based on subject completion of questionnaires or answering a nonleading question by an investigator, with four exceptions [77, 78, 80, 91], one of which involved data mining of medical records with no direct involvement of the subjects [91]. Four of the studies used methodologies that incorporated aspects of observational study design or standardised psychometric testing [77, 78, 83, 90]. The studies are summarised in Table 1. Differences in study design preclude a direct statistical comparison; therefore the adverse event figures are provided merely to illustrate the variation in results.

All ten of the reviewed military studies concluded that mefloquine was safe and well tolerated. One small study designed to compare the efficacy of four different drug regimens found that “mefloquine was well tolerated and no dizziness or neurotoxicity was observed,” while providing no indication in the report as to the methodology underlying that assessment including adverse event reporting [75]. Only one military study [68] used a methodology for adverse event reporting that included standardised psychometric testing. This was a 1993 double-blind RCT involving 359 US Marines that compared two groups taking weekly mefloquine prophylaxis, one of which was given an initial loading dose, to a third chloroquine group. Symptom assessment was conducted using physician interview, Environmental Symptoms Questionnaire (ESQ), and Profile of Mood States (POMS), completed weekly. Sleep and wake cycles were also monitored using actigraph recorders worn by some of the subjects 24 hours a day. The trial was conducted over 12 weeks, with results shown for week 1, weeks 9–12, and overall. Insomnia was a prominent symptom, particularly in the mefloquine loading dose group. There were 10 withdrawals in the mefloquine groups, 6 of which were attributed to insomnia or vivid dreams. Two mefloquine subjects were withdrawn for depression and suicidal thoughts, neither of which was attributed to the drug [78]. In the nonloading mefloquine group, 43% experienced nonsevere neuropsychiatric adverse events including insomnia, 25%, vivid dreams, 7%, dizziness, 6%, headache, 22%, irritability, 4%, poor concentration, 5%, anger, 1%, and moodiness, 1%.

The largest of the military studies [80] is worth specific mention as it exemplifies methodologies for reporting and attribution of adverse effects common to many of the reviewed military studies and contrasts the results of the original study with a follow-up study involving a majority of the original trial subjects [89]. This field study involved 2,289 Dutch military personnel who used mefloquine as weekly chemoprophylaxis while deployed to a United Nations peacekeeping mission in Cambodia in 1992-1993. Adverse events were determined by spontaneous self-reporting, with medical interventions defined as severe adverse events. Possible mefloquine related neuropsychiatric adverse events were reported by 22.8% of the subjects including concentration disorders, 7.8%, dizziness, 5.6%, visual complaints, 2.8%, and insomnia, 1.0%. Of the 2,289 subjects, 7 (0.3%) experienced severe symptoms that they attributed to mefloquine, 5 of which were neuropsychiatric. These included 2 seizures, 1 case of serious myoclonus, and 2 cases of severe dizziness. Not one of these was subsequently attributed to mefloquine by the investigators. One seizure patient had a personal history of epilepsy, but the other did not, and no further events occurred after that subject changed to doxycycline. The myoclonus patient was free of complaints after being changed to doxycycline. Symptoms also ceased in both dizziness patients when their prophylaxis ceased or was modified [80]. A follow-up study asked 1,733 (68%) of the subjects about the symptoms they experienced during their deployment using a mailed questionnaire. Of those 1,733 respondents, 1,638 (95.6%) reported that they had used mefloquine: 49.6% of the mefloquine respondents reported experiencing adverse effects, compared to 12.5% of doxycycline users. In the group that linked their complaints to their deployment, symptoms included vertigo/dizziness, 21.3%; visual complaints, 14.5%; memory loss, 12.7%; fatigue, 12.2%; headache, 11.8%; and concentration problems, 5.4%. Possible explanations offered in the report for the “very high frequency of side effects” include “a widespread mistrust in mefloquine,” suspicion arising from denials on the part of authorities, and recall bias. No clinical observations were made during the study. The report makes no reference to historical evidence of quinoline CNS toxicity [9], previously published case reports linking mefloquine prophylaxis to a variety of the reported symptoms, or evidence of mefloquine accumulation in human brain tissue following prophylaxis [145]. Similar limitations were found in the other military studies, including the ADF trials discussed in Section 7.5.

Several of the civilian studies are worth contrasting with the Dutch military study. The first of these is a 1999 double-blind RCT involving 1013 subjects who enrolled at 15 travel clinics across five countries [76]. Each subject travelled to a malarious area for up to 28 days and then was evaluated at 7, 28, and 60 days after return to obtain information about a targeted list of adverse events and potential malaria episodes. Each investigator assessed whether there was a reasonable possibility that each adverse event was caused by the study drug, without knowledge of which drug the subject had been assigned. An adverse event was treatment emergent if it started while the subject was taking the study drug. Accounting for withdrawals due to changed travel plans and other factors, 966 completed the trial. The two groups were well balanced regarding demographics and other factors. Appropriate controls were implemented to account for varying regimens between the mefloquine group and the comparator group, including placebos. Severe adverse events were defined as those requiring medical advice. Of the 2,120 treatment-emergent adverse events across the entire study population, 1,310 (62%) were considered by the investigator to be unrelated to the study drug. Adverse events attributed to the drug occurred in a significantly higher proportion of subjects who received mefloquine (42% versus 30%) and “the difference was especially pronounced for neuropsychiatric events.” Among subjects who discontinued taking the study drug as a result of an adverse event, the event was attributed to the drug in 37 subjects. Treatment-limiting neuropsychiatric events began in 19 subjects while they were receiving mefloquine, in 5 subjects while they were receiving mefloquine placebo, and in 3 subjects while they were receiving the comparator. No severe adverse events were attributed to either drug; however each is listed in the report and they are clearly not attributable to the drug. In the mefloquine group (*n* = 483) there were 19 nonsevere neuropsychiatric adverse events, including insomnia (12), anxiety (9), strange/vivid dreams (7), dizziness/vertigo (7), depression (3), visual difficulties (3), concentration impairment (3), and other (4). The report compares the results with two other studies to find whether they were consistent [76].

A Danish retrospective study of adverse event reports [90] is of interest not only for its methodology but also for providing an indication of chronic psychiatric effects associated with mefloquine prophylaxis. This study evaluated both acute and long-term psychiatric symptoms in 66 (89%) of 85 individuals who had submitted adverse event reports to the Danish National Drug Authority from 1996 to 2000. Forty of the subjects had complained of more than one symptom in their original adverse event report, with the group experiencing a range of physical/neurological and psychiatric symptoms including anxiety, sleep disturbances/nightmares, depression, possible psychoses (delusions/hallucinations), and cognitive impairment. Acute psychiatric effects were retrospectively assessed using the standard Symptom Checklist-90-Revised (SCL-90-R) psychometric [146] and Present State Examination (PSE) psychiatric [147] tests, with clinically significant scores for anxiety, phobic anxiety, and depression found in 55%, 51%, and 44%, respectively, of the mefloquine subjects. Substantial acute phase psychotic symptoms were found in 15% and were time-limited. Cases of hypomania/mania in the acute phase were found in 5.5% of the mefloquine subjects. Significant long-term mental health effects were demonstrated in the SF-36 Health Survey [148] subscales of mental health (MH), role emotional (RE), and vitality (VT) in the mefloquine group compared to control groups matched by age and gender [90].

Methodological limitations identified in many of these pharmacoepidemiological studies tend to reinforce previous findings of a systemic underreporting of adverse events [73, 74].

However, the reported incidence of adverse neuropsychiatric effects has continued to increase over time. For example, several studies published in the early 2000s reported an incidence of symptoms such as nightmares, anxiety, and psychosis that were at least 100 times higher [74, 76, 77] than was reported in the early 1990s [8, 10, 74]. More recently, the Australian manufacturer's 2014 product information shows an incidence of anxiety, depression, suicidal ideation, and encephalopathy [12] ten times higher than the 2013 edition of the same document [149]. As the literature on pharmaceutical RBA would suggest [34, 35, 37, 39], these more recent figures provide a better understanding of the incidence of mefloquine's neuropsychiatric adverse effects than initial “crudely calculated” [16] estimates made soon after the drug's introduction, which included an explicit caveat that “the real incidence of side effects may be greater than that revealed in our study” [92], in the absence of more appropriate phase III clinical trials [14]. What some of the studies examined above [77, 78, 83, 90] do illustrate however is the utility of observational study design including standardised neuropsychometric testing where this can be appropriately incorporated into pharmacoepidemiological studies.

### 5.4. Experimental Neuropharmacology

Early in mefloquine's development the drug was found to have a long elimination half-life relative to other quinolines and classes of antimalarials [150], of approximately two to four weeks [151]. This property gave it an advantage over other drugs in the search for alternatives to defeat chloroquine-resistant* Plasmodium* in that a prophylaxis regimen of less frequent doses might also offer improved compliance [152, 153] or cost effectiveness [13] relative to those requiring a daily dose. A dose of 250 mg once per week was initially recommended; however concerns that toxic accumulation may occur during weekly administration for long-term chemoprophylaxis led to early recommendations for a dose of 250 mg every second week during long-term use [17]. Failure rates in some groups and subsequent pharmacokinetic investigation [154] saw that 250 mg per week became the standard in the US [17]. The pharmacokinetic study cited by the CDC in recommending this change monitored plasma levels in 15 adult subjects for 13 weeks to find that “toxic accumulation does not occur” at peak levels under a weekly regimen. The report mentions that each subject was given a diary for recording doses and adverse effects and then interviewed by the investigator at the conclusion of the study, making no mention of any adverse effect reporting results [154].

The year following publication of the above pharmacokinetic study, evidence of mefloquine accumulation in postmortem human brain tissue linked to prophylaxis was published [145], and in 1997 the drug was demonstrated to cross the blood-brain barrier in animal models [155]. Researchers associated with WRAIR recognized in 2004 that mefloquine's clinical potential may be compromised by neurotoxicity [156]. As a small, lipophilic molecule [157, 158], the drug is easily able to cross the blood-brain barrier [16, 155, 157], accumulate in the CNS, and interact with neuronal targets [19, 20, 159, 160] including within limbic system and brainstem [8, 9, 19]. The drug's precise biochemical mechanism of action in causing lasting CNS neuronal injury is yet to be determined [157]. The drug is known to interfere with normal gap junction functioning [22, 23, 159, 161]; and the series of studies that first demonstrated its neurotoxicity [19] continued to investigate mefloquine's ability of disrupting calcium homeostasis and perturbing the endoplasmic reticulum [20–23], which is a known causal mode of neuronal cell apoptosis [162, 163].

One remaining area of debate is the dose-dependent incidence of neuropsychiatric effects. While there is caution regarding a higher risk of toxicity with treatment doses [11] and the US FDA has warned of a risk of lasting or permanent CNS effects at prophylactic doses [113] the prevailing view has been that any acute effects will cease once the dosing is discontinued and the drug is eliminated [12]. The WHO/F. Hoffmann-La Roche report of 1991 which addressed this issue of dose dependence stated that, although adverse events “seem to be more frequent when higher doses are used,” based on the evidence then available, “there [was] no compelling evidence that CNS reactions associated with mefloquine are dose-dependent” [16]. Almost a quarter of a century after that report was published, evidence now suggests that doses associated with mefloquine prophylaxis, not only those used for treatment of malaria, can cause lasting CNS injury with chronic sequelae [8, 9]. The seminal 2006 study that found mefloquine to be neurotoxic equated doses that were used during that study to elicit toxicity-induced behaviours “that are similar to those observed in humans after the treatment [vice prophylaxis] dose” [19]. However mefloquine levels measured in the brain of individuals who were taking the drug acutely (750 mg, 37 to 70 hours before death) were found to be 51.5 nmol/g [164], which equates to plasma levels of approximately 137 nM [165]. The prophylaxis-related brain tissue concentrations of 8.7 to 14 mg/kg found in patients examined in the postmortem study cited above [145] also translate to serum levels of 100 to 135 nM with humans undertaking a long-term prophylaxis suggested to have even higher tissue levels [165]. Together, these studies suggest that treatment with mefloquine at prophylactic levels can give rise to drug concentrations in the brain sufficient to cause CNS toxicity, previously presumed to equate only to higher treatment doses [19].

One of mefloquine's important characteristics, related to the question of dose dependence, is the idiosyncratic nature of its neuropsychiatric reactions [9]. Well-known idiosyncrasies with other antimalarial quinolines have been fundamental to drug safety in global malaria eradication programs, for example, haemolytic anaemia in primaquine patients with glucose-6-phosphate dehydrogenase (G6PD) enzyme deficiency [166]. Although at least one author has hypothesised as to how some mefloquine users may be genetically predisposed to some of the drug's adverse effects [167] and it is known that the US Army approached private industry in part to “identify genetic and other predispositions to mefloquine neurotoxicity” almost a decade ago [112], the present review was unable to identify a research program dedicated to investigating this aspect of the drug's properties. Absent such research, an assumption that adverse reactions to mefloquine are necessarily dose-dependent or attributable to a preexistence of latent psychiatric illness [3, 12] appears to be no longer sound.

### 5.5. Confounding Diagnosis of Prevalent Neuropsychiatric Illnesses

Two neuropsychiatric conditions relevant to the use of mefloquine in military populations are PTSD and TBI. Due to their prevalence and overlapping symptomology there is extensive literature on comorbidity and differential diagnosis between those conditions [168–170]. As the understanding of mefloquine neurotoxicity and its prevalence has grown in recent years, attention is now being drawn to the relationships between mefloquine and those two conditions, with the US Centers for Disease Control now advising that mefloquine's “neuropsychiatric side effects may confound the diagnosis and management of post-traumatic stress disorder and traumatic brain injury” [10, 25].

PTSD is a psychiatric disorder that can result from exposure to trauma, where the exposure comprised an actual or threatened death, serious injury, or sexual violence [171]. Until recently diagnostic criteria for PTSD did not exclude symptoms resulting from direct effects of medications. This means that patients experiencing mefloquine neurotoxicity may have appeared to meet PTSD diagnostic criteria regardless whether their symptoms were caused by traumatic stress. Many of mefloquine's reported adverse neuropsychiatric effects are consistent with key PTSD diagnostic criteria including “intrusion or reexperiencing” (Criterion B), “negative alterations in mood or cognitions” (Criterion D), and “increased arousal symptoms” (Criterion E) and may be persistent (Criterion F) in cases of long-term or permanent neuronal injury [10].

TBI, which involves brain damage caused by external force, has received widespread attention in recent years due to the exposure of military personnel to blast injuries in Iraq and Afghanistan [172]; however it is more commonly caused by falls, sports, and motor vehicle accidents [173]. The injury can result in persistent symptoms, or even postconcussive syndrome (PCS), including somatic complaints, depression, anxiety, personality disorders, and cognitive impairment [174]. As yet there are no published studies regarding differential diagnosis between mefloquine neurotoxicity and TBI; however the overlapping symptomology does suggest a prospect of misdiagnosis in cases where there has been no obvious physical trauma and/or the symptoms are relatively mild.

TBI is frequently comorbid with PTSD and there is evidence that even mild TBI (mTBI) can increase risk for PTSD and other psychiatric conditions. There is debate that postconcussive sequelae including psychiatric disorders and cognitive impairment secondary to mTBI may be attributable to either psychological stress or neurobiological injury, with some authors favouring psychological treatments in cases where the cause is not neurobiological [168, 169]. Although a variety of neuropsychological and neuroimaging methods are available to assist in differential diagnosis between PTSD and TBI [170], the microscopic and highly focal nature of neuronal degeneration associated with mefloquine neurotoxicity is likely undetectable by conventional neuroimaging [9].

### 5.6. Synthesis

Having reviewed the previously published research, it is now possible to synthesize the various findings related to mefloquine CNS toxicity. Given the time-limited nature of most of the pharmacoepidemiological studies conducted to date, overall they cannot reflect a prevalence of a* chronic* mefloquine CNS toxicity syndrome. One exception is a recently published study finding long-term mental health impacts on individuals who had previously reported acute or subacute adverse effects [90]. The literature on neurotoxic encephalopathies and syndromes indicates that many typical symptoms may be subclinical, not easily recognized by the patient, and nonspecific and mimicked by other neuropsychiatric diseases, with longer exposures to neurotoxic agents more likely to result in a diagnosable illness [45, 46, 49, 50]. The various manifestations of mefloquine CNS toxicity [8, 9] are consistent with a variety of chronic psychiatric and neurological diseases independently determined to be causally related to mefloquine use, based on medical-scientific evidence in accordance with epidemiological practice [93, 131–141, 175], although in some cases mefloquine CNS toxicity provides a more plausible mode of action [9]. Despite findings of systemic underreporting of adverse events [73, 74], the manufacturer now states that some of these overt disorders, or associated symptoms, are common among mefloquine prophylaxis users [12]. In the absence of appropriately scaled, inclusive, longitudinal, neurotoxicology studies demonstrating otherwise, this evidence suggests that a chronic CNS toxicity syndrome associated with mefloquine prophylaxis may in fact also be common.

## 6. Neuropsychiatric Illness in the Australian Defence Force

### 6.1. Prevalence and Research

The prevalence of neuropsychiatric illness in ADF serving personnel and veterans, including suicide, has recently been the subject of extensive study. A 2010 study estimates that 54.1% of the population of just over 50,000 ADF personnel experience psychiatric disorders in their lifetime, including 4,757 (20.8%) with affective disorders and 7,420 (27%) with anxiety disorders. Within the preceding 12 months only, the respective figures were 9.5% for affective disorders and 14.8% for anxiety disorders. No significant difference was found in the prevalence of these disorders between personnel who had deployed on operations and those who had never deployed [26]. Nonoperational trauma in the ADF, including bullying and sexual abuse, has also been studied extensively [176].

The 2010 study found that the prevalence of suicide ideation was “significantly higher in the ADF compared to the community,” although the study does note that ADF members are less likely to complete the act of suicide. Significantly, only half the sample with PTSD or depressive episodes reported receiving treatment in the previous 12 months, due to a variety of barriers including stigma. The study analysed factors such as trauma exposure, caffeine and tobacco use, alcohol and illicit drug abuse, and use of dietary supplements; however prescription drugs were not considered [26].

A 2009 independent study [94] was undertaken specifically to examine suicide among Australian veterans. This study did consider prescription drugs, but only the role of antidepressants in suicide prevention. Abuse of illicit drugs was also considered. One key section of the report identifies “risk factors for suicide that can be of use when planning prevention strategies,” citing research that provides “a detailed assessment of the strength of evidence for risk factors associated with suicide in the general population,” including “Level A evidence [that] is strong evidence with conclusive results” [94]. List A from the report is reproduced in full as follows. 

*“Level A” Risk Factors for Suicide (Adapted from [94], Emphasis Added)*

1. Demographic factors: males aged 30–34, indigenous, rural, and remote populations.
2. Psychopathology and psychiatric hospitalisation:* diagnosis of a mental disorder*, particularly* affective disorders*, substance abuse,* anxiety disorders*,* personality disorders*, and* psychiatric comorbidity*.
3. Previous nonfatal suicidal behaviour and* suicidal ideation*.
4. Family history of psychopathology and suicidal behaviour.
5. Physical illness, chronic physical pain.
6. Negative life events and low coping potential.
7. Marital status of divorced, widowed, or separated.
8. Low socioeconomic status, unemployment.
9. Neurobiological activity:* hypoactivity of the serotonergic system* (mefloquine is a 5-HT3 receptor antagonist (serotonin blocker) [177, 178]).
10. Psychological factors: hopelessness;* high aggression and impulsivity*, lack of reasons for living,* cognitive rigidity*, low ability to solve problems, perfectionism, and* psychological pain*.
11. Social isolation and lack of social support.

Aspects of the four (of 11) factors linked to mefloquine use are emphasised above in italics. In relation to the psychiatric disorders listed in factor (2), the manufacturer currently advises that anxiety and depression are common (≥1/100 to <1/10), while hallucinations, bipolar disorder, and psychotic disorders including delusional disorder, depersonalisation, and mania are uncommon (≥1/1,000 to <1/100). In relation to factor (3), the manufacturer currently advises that suicidal ideation is uncommon (≥1/1,000 to <1/100). In relation to factor (9), mefloquine is a known 5-HT3 receptor antagonist [177, 178]. In relation to factor (10), the manufacturer currently advises that agitation, restlessness, mood swings, panic attacks, confusional state, aggression, depersonalisation and mania, and paranoia are uncommon (≥1/1,000 to <1/100) [12].

### 6.2. Policy Responses

The above research findings have resulted in significant reforms to mental health and related policies by the ADF and Department of Veterans Affairs (DVA). A 2009 review of mental health care in the ADF recommended a series of reforms, including improved governance and policy, improved training, enhanced rehabilitation and transition services, and greater involvement of families [27]. The resulting 2011 ADF mental health strategy emphasizes the ADF's commitment to “evidence-based treatment and recovery programs” and “innovation and research that improves our understanding of mental health and wellbeing,” through key objectives such as “identification and response to the mental health risks of military service” and “building an evidence base about military mental health and wellbeing” [5]. The 2013 DVA veteran mental health strategy includes similar objectives, such as “strengthening workforce capacity” and “building the evidence base” [179]. Prior to these reforms, existing ADF policies already subjected personnel to mandatory periodic, pre- and postdeployment mental health screening [28].

### 6.3. Posttraumatic Stress Disorder

The 2010 ADF mental health study cited above estimated that 8.3% of ADF personnel experienced PTSD in the preceding 12 months [26]. ADF health policy states that its personnel are considered to be a high risk group due to their exposure to traumatic events associated with operational deployments and that exposure to further stressors should be limited for those suffering the condition. The same policy states that PTSD is often comorbid with mTBI and notes that neuropsychological testing should be undertaken when mTBI is suspected. Treatment should be evidence-based, and the policy endorses trauma-focused cognitive therapy and/or pharmacological therapy as required [29].

### 6.4. Traumatic Brain Injury/Postconcussive Syndrome

Detailed data on the prevalence of TBI in the ADF is not publicly available. However in the Australian community the prevalence of mTBI has been estimated at 64–131 cases per 100,000 population each year, with moderate and severe TBI at 15–20 per 100,000 and 12–14 per 100,000, respectively. Prevalence is highest in the 15–35-year age group and significantly more common in males than females by a ratio of 3-4 : 1. Common causes include falls, sport, and motor vehicle accidents [173, 174]. ADF health policy notes that one of the signature symptoms of TBI is cognitive impairment. This presents considerable risk in that “cognitive tasks such as safe driving, handling firearms, establishing situational awareness and the ability to control aggression may result in adverse outcomes such as friendly fire incidents.” Specific measures to manage risks associated with TBI include the use of protective equipment and mandatory neurocognitive baseline testing for all personnel prior to operational deployments [30].

## 7. Mefloquine RBA in the Context of ADF Neuropsychiatric Illness

### 7.1. Organisational Context

The use of mefloquine and other drugs for malaria prophylaxis in healthy people is in itself a risk reduction method, where the benefit to the individual is the prevention of a serious disease and in the case of a military organisation reduces the costs and further risks associated with treating, managing and evacuating patients, and resulting loss of military capability. Beyond the narrow context of malaria prevention, however, the broader military context highlights key additional risks to both individuals and the organisation. Given the ADF's focus on operational stress and mental health since at least the mid-1990s, [4–6] sound RBA relating to mefloquine use would have considered not merely the direct, individual risk of adverse effects but secondary and organisational risks such as complicating the diagnosis and treatment of other prevalent conditions with similar symptomology.

Although ADF RBA relating to mefloquine use is not publicly available, policies of using the drug as an alternative to contraindicated prophylaxis and prohibiting use by specialist personnel, citing the neuropsychiatric adverse effects, are apparent risk reduction measures. Viewed purely within a context of malaria prevention this RBA approach appears adequate. However evidence of the neuropsychiatric adverse effects since the drug's inception and the comorbidity of neuropsychiatric illness in the ADF would warrant a more comprehensive RBA including several other key factors. These include identification of long-term risks, barriers to recognition and reporting of adverse drug effects, duration and repetition of exposures, conduct of clinical trials in a military setting, and ongoing risk monitoring and management. Each of these factors is examined below, in relation generally to mefloquine use in the ADF and more specifically to its use in clinical trials that comprised a large proportion of the overall risk exposure to ADF personnel.

### 7.2. Identification of Long-Term Risks

Policies on health and risk management in the ADF emphasize an “evidence-based” approach to management [1, 5]. The ADF has been directly involved in research into the quinolines since the 1940s [115–118, 180] and mefloquine specifically since at least 1988 [85–88, 106, 107], with AMI having had a long association with WRAIR and other organisations that have studied the drug's toxic properties [15, 85–88, 106, 107, 128, 129]. Mefloquine's safety profile has been characterised by adverse neuropsychiatric effects since its introduction [16]. Clinical evidence of toxic encephalopathy linked to mefloquine use was published as early as 1987 [60]. Direct evidence of mefloquine accumulation in human brain tissue following prophylaxis was published in 1994 [145]. A meta-analysis of mefloquine studies that found that the drug “has adverse effects that limit its acceptability” was published in 2000 [73]. Direct evidence of neurotoxicity was published in 2006 [19] as part of a series of published studies conducted by researchers associated with WRAIR [20–23], prompting the US Army to solicit industry proposals “to define the biological mechanisms of mefloquine neurotoxicity” [112]. The RMA has made a series of independent determinations that mefloquine and other quinolines can cause a variety of psychiatric [131–134], neurological [135, 136, 138–140], vestibular [137], and cardiac diseases [141], similar to a list included in a 2004 US Department of Veterans' Affairs document raising concerns regarding the long-term health impacts of mefloquine use [142]. Therefore it is reasonable to conclude that the recent findings relating to mefloquine neurotoxicity [19–23] as a cause [8, 9] or significant confounding factor in chronic neuropsychiatric illness [9, 10, 25] were foreseeable.

The ADF did apparently mitigate the risk of more widespread exposure by limiting the general use of mefloquine to a second- or third-line agent [12, 85, 86, 106, 107]. However it is not apparent that the organisation recognized the above evidence by assessing the longer-term risks of complicating the health management of personnel who have previously been exposed to mefloquine. Despite the ADF's involvement in a number of longitudinal neurotoxicological studies to assess the exposure of other specific populations to environmental and medical toxic agents [42, 178], no such studies into the health impacts of mefloquine use have been conducted.

### 7.3. Barriers to Recognition and Reporting of Adverse Drug Effects

Sound RBA involving significant adverse drug effects would include critical analysis of any barriers to recognition and reporting of those effects by patients, trial subjects, and health practitioners. In the case of mefloquine use in military settings there are at least several barriers that result from the context of the environment and perceptions of mefloquine users and health practitioners. Firstly, many of mefloquine's documented neuropsychiatric effects are not reasonably distinguishable from normal psychological or physiological reactions to psychological or environmental stressors prevalent in military settings where the drug is used. Well-documented psychological stressors include danger of being killed or maimed, exposure to trauma, loss of sleep, long duration of deployments, and separation from family [4, 7], while physiological stressors include exposure to extreme temperatures, loss of sleep, fatigue, disease, poor air and water quality, noise, vibration, and toxic materials [7]. Many neuropsychiatric symptoms linked to these prevalent stressors, including depression, anxiety, headache, and dizziness [4, 7, 181, 182], are also reported by the manufacturer to be common effects of mefloquine [12].

This operational context suggests that personnel who experience acute symptoms may be more likely to endure them or attribute them to other prevalent environmental factors than report them as adverse drug effects. In cases where chronic symptoms persist after a deployment in which mefloquine was used, there are additional relevant considerations. Typically, cessation of mefloquine chemoprophylaxis would coincide with an individual's departure from the stressful operational environment described above. The manufacturer's advice that any adverse effects would cease once the drug is discontinued would tend to reinforce an individual's tendency to attribute any persistent symptoms to other factors. Further, when ADF personnel depart an operational area they are required to complete a general health questionnaire that prompts them to record their exposure to “hazardous situations,” including many of the stressors listed above [183, 184]. However prescription medications are not listed on this documentation, so this process in itself would tend to result in a bias towards attributing any symptoms to the “officially acknowledged” exposures. A second barrier is the well-documented stigma of reporting including concerns by individuals seeking treatment for neuropsychiatric symptoms among military personnel [26, 185–187]. Stigma for reporting neuropsychiatric illness identified in current ADF mental health doctrine includes concerns by individuals that they would not be deployable (or, by extension, removed from a current deployment), that they would be treated differently by other people, or that their careers would be adversely affected [6].

A third barrier is that a common symptom of the neuropsychiatric conditions associated with mefloquine [9, 12] is cognitive impairment [110]. The capacity of an individual to recognize symptoms that are already difficult to distinguish from normal reactions and already attract stigma would clearly be further diminished by cognitive impairment. An expectation that an individual experiencing cognitive impairment would identify and report the symptoms of cognitive impairment would be perverse. These barriers to reporting both acute symptoms while taking mefloquine or chronic symptoms after cessation exacerbate risk by reducing reporting during drug trials or submitting adverse reports to drug regulators and reduce the likelihood of personnel with chronic conditions from receiving subsequent care.

### 7.4. Duration and Repetition of Exposures

Mefloquine use has coincided with a period of high operational tempo for the ADF. In 2010 the ADF population had a mean length of service of 11.6 years. An estimated 43% had experienced multiple overseas operational deployments, ranging from four to 12 months [26]. Although figures on mefloquine use are not publicly available, a large proportion of ADF personnel were deployed to malarious areas where they were also exposed to the other stressors identified above. Notably, the risk of developing PTSD, TBI, and other neuropsychiatric illnesses is not exclusive to operational deployment and many personnel may not seek treatment, leaving them predisposed to additional stressors. While it may be true that adverse mefloquine reactions can be attributed to preexisting neuropsychiatric illness in some cases, the reverse may also be true in others that exposure to mefloquine toxicity could predispose individuals to other prevalent neuropsychiatric disorders. Given the duration and repetition of these combined exposures, this warrants identification of mefloquine use in an individual's history to aid in correct diagnosis and subsequent care for neuropsychiatric patients.

### 7.5. Conduct of Clinical Trials in a Military Setting

The AMI has conducted several trials involving mefloquine use by ADF personnel as trial subjects [85–88]. Two of these involved personnel deployed on peacekeeping operations in East Timor during the period 2000–2002, totalling more than 1,300 mefloquine recipients [87, 88]. By that time the drug had been on the market for approximately a decade and concerns regarding its neuropsychiatric effects were prominent [14, 15, 17], with doxycycline used as the first-line prophylaxis and mefloquine as the second line [3, 15, 86–88, 106, 107]. The current international standard for good clinical practice in pharmaceutical trials had been mandated by the Australian government's health and medical research statutory body in 2000 [95]. Several key aspects of that standard are relevant to the conduct of the trials by AMI in a military setting. Firstly, the standard describes “members of the armed forces” as* vulnerable subjects* “whose willingness to volunteer in a clinical trial may be unduly influenced by the expectation, whether justified or not, of benefits associated with participation, or of a retaliatory response from senior members of a hierarchy in case of refusal to participate.” Secondly, “foreseeable risks and inconveniences should be weighed against the anticipated benefit for the individual trial subject and society.” Thirdly, the standard states that “the rights, safety, and well-being of the trial subjects are the most important considerations and should prevail over interests of science and society.” Fourthly, “during and following a subject's participation in a trial, the investigator/institution should ensure that adequate medical care is provided to a subject for any adverse events, including those related to the trial.” Finally, in obtaining the informed consent of trial subjects, the institution should adhere to “the ethical principles that have their origin in the Declaration of Helsinki” [95].

The first trial [87] was a phase III RCT for the safety and efficacy of tafenoquine prophylaxis, with mefloquine used as the comparator drug, including a prophylaxis phase and a follow-up treatment phase. 492 subjects received tafenoquine and 162 received mefloquine. Adverse event reports were elicited by the investigator asking the nonleading question “do you feel differently in any way since starting the new treatment?” Severity and attribution to mefloquine prophylaxis were then determined by a study physician. No standardised neuropsychometric testing was conducted. In the mefloquine prophylaxis group, 143 (88.3%) of the subjects reported at least one adverse event, 23 (14.2%) of whom reported neuropsychiatric adverse events: 19 (11.7%) mefloquine subjects experienced adverse events with a “suspected/probable” relationship to prophylaxis. Three (1.9%) mefloquine subjects withdrew from the study as a result of adverse events, with those 3 subjects reporting 5 (3.1%) adverse events between them. There are ambiguities in the report regarding the withdrawals. A table in the report shows that 4 (2.5%) mefloquine subjects withdrew from the trial due to adverse events, 4 (2.5%) changed to other antimalarial drugs, and 1 (0.6%) withdrew for a reason unrelated to the trial, leaving 153 (94.4%) to complete the trial. The text of the report states that there were 3 (2%) severe adverse events experienced by mefloquine subjects, but none of these were neuropsychiatric. The report does not state whether any of the mefloquine subjects who withdrew from the trial or changed to other drugs experienced nonsevere neuropsychiatric adverse effects. A further ambiguity is found in the abstract, which states that “Three subjects on tafenoquine (0.6%) and none on mefloquine discontinued prophylaxis because of possible drug-related adverse events.” This suggests that the investigator did not attribute the adverse events experienced by the withdrawn subjects to mefloquine use, although that is not stated in the report. Regardless of these ambiguities, the nonsevere adverse neuropsychiatric events experienced by the mefloquine group included vertigo, 8 (5%); somnolence, 6 (4%); abnormal dreams, 2 (1%); dizziness, 2 (1%); insomnia, 3 (2%); abnormal coordination, 1 (0.6%); and depression, 1 (0.6%). The report found that “mefloquine was well tolerated by the Australian soldiers, which is in accordance with the results of other randomized, double-blind studies of military populations,” citing two trials which are summarised in this review [78, 81]. Eventually published some eight years after the trial in 2010, by which time WRAIR had established a permanent research laboratory at AMI [129], the report makes no reference to the fact that mefloquine had been found to be neurotoxic [19].

The second trial [88] was an open-label, prospective study to describe the tolerability of mefloquine malaria prophylaxis in comparison to doxycycline. Of the study subjects, 1,157 were administered mefloquine on the rationale that “there are limited data on the tolerability of mefloquine for long-term prophylaxis in military personnel.” Participation was claimed to be voluntary, with nonvolunteers using doxycycline [88]; however it has since been reported that the commanding officer of approximately half of the subjects directed his subordinates to participate under threat of being excluded from the deployment [188, 189]. Mefloquine subjects included 75 (6.5%) who withdrew from the trial due to adverse events attributed to the drug, including 62 (5.3%) who withdrew due to neuropsychiatric adverse events. At least one adverse event was reported by 57% of the subjects. There were three severe neuropsychiatric adverse events “possibly relating to mefloquine.” One of these three subjects “experienced depression, episodic anxiety, mild paranoia, short-term memory loss and suicidal ideation” and his “mental state continued to deteriorate” despite ceasing mefloquine. Only preliminary figures are reported for nonsevere adverse events. In the discussion, the report states, “when monitoring the tolerability of a drug under military operational conditions, there is a need to account for the physiological and psychological stress associated with such activities that may confound the relationship between drug intake and adverse events.” The trial report concluded that mefloquine was “well tolerated” by the subjects and simply recommended that it “continue to be used for those intolerant of doxycycline” [88]. In 2004, approximately one-quarter of the mefloquine subjects initiated legal action against the ADF and the manufacturer, reporting that they were not adequately informed of side effects and complaining of symptoms such as depression, paranoia, and suicide ideation [190].

Given the clinical standards quoted above [95], it is difficult to conclude that these trials were ethical or that their resultant findings as to the tolerability of mefloquine are valid. While the trial reports state that the subjects were properly informed volunteers, one-quarter of them subsequently initiated legal action on the basis that they were not [190] and there is no mention of this in the published reports even though the reports were published after the legal action was initiated. There is further evidence that as many as half of them were unduly influenced to participate in the trials [188, 189]. The safety and well-being of the subjects were placed at risk for no appreciable benefit, as mefloquine was already licensed and was being used only as a second-line drug in recognition of its neuropsychiatric safety risk. Although both reports analyse neuropsychiatric adverse events, there is no analysis of the barriers to reporting described above, either during or subsequent to the trials [87, 88]. One of the reports does note the limited external validity of the trial; however this observation relates to gender rather than the military operational setting of the trial [88]. From a RBA perspective, the trials exposed the participants to significant risk with no appreciable benefit.

### 7.6. Ongoing Risk Monitoring and Management

RBA is not static but comprises part of a continuing, dynamic risk management process that should logically not only extend through a drug's lifecycle but also address any subsequent adverse outcomes. Recognition that historical mefloquine use poses a higher risk than was earlier appreciated therefore warrants an introduction of additional risk management measures, beginning with the identification and screening of previous mefloquine users. The ADF already has standard procedures for reducing risks associated with malaria and neuropsychiatric illness. For example, in order to minimize the risk of haemolytic anaemia caused by ADF's terminal prophylaxis drug, primaquine, all ADF personnel are tested for G6PD deficiency, with the results recorded in their health records [3]. Risk management measures for neuropsychiatric illness include the general health screening, mental health screening, and neuropsychological baseline testing cited above.

Given that mefloquine is a prescription drug, now recognized as a factor in neuropsychiatric illness that can confound diagnosis of prevalent neuropsychiatric conditions, and noting the above barriers to recognizing symptoms of the drug's chronic effects, similar screening for mefloquine recipients would be a prudent risk reduction measure. Screening for mefloquine neurotoxicity could begin by identifying users from pharmaceutical records and include neurological vestibular function and neuropsychological cognitive function tests for those identified. These would assist investigation, correct diagnosis, and differential diagnosis, not only improving the management of other prevalent illnesses but also reducing the risk of misdiagnosis and subsequent mistreatment. For example, pharmacotherapy presents an array of possible adverse effects [191]. Despite misconceptions to the contrary, trauma-focused psychotherapies can cause adverse effects such as depression, panic attacks, suicide ideation, and substance abuse relapse, even when found to be efficacious in correctly diagnosed patients [192, 193]. Minimizing extraneous exposure to these therapies reinforces the case for proactively identifying and screening mefloquine recipients to aid correct diagnosis, rather than relying on self-reporting of psychiatric symptoms. A further measure would then be developing guidelines to assist clinical care providers in identifying personnel affected by mefloquine neurotoxicity, conducting differential diagnosis with other prevalent conditions, and providing ongoing care and management.

## 8. Conclusions

Both positive and negative conclusions can be drawn from the experience of mefloquine use in the ADF in an RBA context. The individual and organisational benefits of chemoprophylaxis as a measure for preventing the serious illness of malaria are well established, including the use of alternatives for personnel contraindicated for first-line drugs. Risks arising from general mefloquine use since its introduction in the ADF have evidently been reduced via policies that limited its use as a second- or third-line malaria prophylactic and prohibiting its use by specialist personnel, explicitly citing the drug's acute neuropsychiatric adverse effects among other factors.

There are two negative conclusions. Firstly, the particular use of mefloquine in clinical trials, involving large numbers of personnel in a military operational setting, contrary to relevant guidelines, represents an apparent failure to identify the foreseeable risk of causing or aggravating neuropsychiatric illnesses prevalent in the military population from which the trial subjects were drawn. Secondly, the ADF did not appropriately monitor the risks of mefloquine use as insights into the drug's neurotoxicity, the chronic nature and frequency of its neuropsychiatric adverse effects, and its ability to confound the diagnosis of other prevalent illnesses were revealed by the manufacturer and independent research. Nor has the ADF subsequently managed those risks by implementing appropriate measures to care for affected personnel. In effect the mental health, medical and social costs have thus far been transferred to patients and other members of society.

The full extent to which mefloquine use in the ADF has complicated the already difficult problem of mental health management is not yet known and may never be. At best, it has complicated the diagnosis, treatment, and management of neuropsychiatric illnesses prevalent in the target population including PTSD and TBI. At worst, it may have caused or aggravated neuropsychiatric illness in large numbers of patients who have subsequently been misdiagnosed, mistreated, or otherwise failed to receive proper care, despite mental health being a major focus of recent ADF research and policy reform. These risks were foreseeable and should have been considered by health officials during RBA, policy decisions, and ongoing risk management.

The case of mefloquine use in the ADF also provides a useful insight into the interpretation of quantitative versus qualitative evidence by researchers, policy makers, and clinicians in drug safety, particularly prophylactic drugs where alternative drugs and other preventive measures are available. Perceptions of mefloquine as a “safe” drug have emanated from an uncritical bias towards quantitative evidence suggesting that the incidence and severity of the drug's neuropsychiatric adverse effects were relatively low, attributable to other factors such as preexistence or predisposal to psychiatric illness, or merely transient until prophylaxis has ceased. More prudent RBA would have better considered the qualitative evidence indicating that mefloquine concentrations equivalent to those achieved in human prophylaxis are able to cause lasting injury to the CNS with chronic sequelae, thereby compounding the risks of neuropsychiatric illness already prevalent in the population.

Notwithstanding this specific focus on the ADF, a key finding of this review is that there is now compelling evidence for the previously described chronic CNS toxicity syndrome [8, 9], linked to mefloquine prophylaxis, consistent with the literature on epidemiology and neurotoxicology. There is direct evidence that mefloquine is able to accumulate in the human brain and interact with neuronal targets in the CNS, consistent with both clinical observations and plausible pathophysiological mechanisms. There is direct evidence that mefloquine concentrations equivalent to human prophylaxis are able to cause lasting or permanent injury to neurons in animal models, eliciting behavioural responses consistent with equivalent behaviours observed in human prophylaxis users and published in case reports. Multiple neurological syndromes occur in response to this single neurotoxic agent, including a variety of chronic, clinical disorders which have been determined as causally linked to mefloquine use by competent medical authorities, in accordance with epidemiological principles, against an appropriate standard of medical-scientific evidence. The syndrome has varying presentations involving a host of nonspecific symptoms. The symptoms are often mimicked by other prevalent neuropsychiatric disorders, with competent medical authorities having determined that mefloquine use is able to confound the diagnosis and management of those disorders. This now places an onus on public and military health officials to conduct appropriate longitudinal neurotoxicology studies, further medical research, and develop clinical guidelines necessary for the proper diagnosis, care, and management of those affected. This would not only ensure adequate care, but also mitigate the continued risk of administering contraindicated treatments. Given that many of those affected by mefloquine neurotoxicity are veterans or serving members of the military, one might reasonably expect such endeavours to be afforded a high priority for funding and a certain degree of urgency.

In a complex organisation such as the ADF, with large numbers of personnel exposed to a wide range of complex health threats, sound risk management necessitates the inclusion of multiple fields of expertise to identify, assess, and mitigate risk. Shortcomings in RBA identified in the present review appear to have resulted from a bias towards prevention of a serious but well-known disease, drawing narrowly on expertise resident in one specialist research institution, focusing on the beneficial effects of a drug without critically analysing its significant adverse effects. This continued even as the ADF health system was undergoing major reforms to implement evidence-based mental health strategies. With benefit of hindsight, such bias may have been avoided had ADF senior health officials adopted a more inclusive, comprehensive approach, incorporating the fields of neurology, toxicology, psychiatry, psychology, and epidemiology to identify, assess, and mitigate risks as they became evident in research from those fields. The necessity of a critical, inclusive, interdisciplinary approach to organisational health management and risk management is a salient lesson for general and specialist health practitioners, researchers, and policy makers alike.

## Figures and Tables

**Table 1:** Summary of selected pharmacoepidemiological studies relating to mefloquine prophylaxis safety and tolerability.

Year	Reference	Country	Population	Study design	Nonsevere adverse event (AE) report	Standardised testing^1^	Participants	Severe AE (%)^2^	Nonsevere AE (%)^2^
1993	[85]	Australia	Military	Nonrandom field trial	Unknown	Nil	40	n.d.	n.d.

1993	[78]	US	Military	RCT	Questionnaire, interview	POMS, sleep monitoring, and ESQ	203	0%	43%

1996	[75]	Australia	Civilian	Retrospective	Questionnaire (mail)	Nil	285	0%	6.3%

1996	[80]	Netherlands	Military	Nonrandom field trial	Questionnaire (mail)	Nil	2,289	0%	22.8%

1996	[83]	Switzerland	Civilian	Longitudinal	Investigator nonleading question	POMS, NES, and ESQ	420	0%	7.9%

1997	[81]	UK	Military	Nonrandom field trial	Questionnaire	Nil	317	0%	29.0%

1999	[82]	Italy	Military	Retrospective	Questionnaire	Nil	1,386	0%	17.0%

2001	[76]	Neth., Ger., UK, Can., and SA	Civilian	RCT	Questionnaire, interview	Nil	483	0%	3.9%

2002	[77]	Netherlands	Civilian	RCT	Screening, interview	POMS, NES	58	n.d.	n.d.

2005	[88]	Australia	Military	Nonrandom field trial	Questionnaire	Nil	1,157	n.d.	n.d.

2005	[91]	Canada	Military	Retrospective	Data-mining medical records	Nil	1,413	n.d.	n.d.

2007	[79]	Japan	Military	Nonrandom field trial	Questionnaire	Nil	1,876	0%	18.2%

2008	[84]	Sweden	Military	Retrospective	Questionnaire	Nil	488	0%	57%

2010	[87]	Australia	Military	Nonrandom field trial	Investigator nonleading question	Nil	162	0%	11.7%

2014	[90]	Denmark	Civilian	Retrospective	AE report to drug regulator	SCL-90-R, PSE, and SF-36 (long-term)	67^3^	n/a^4^	n/a^4^

Total							10,664		

*Notes*.

^1^POMS: Profile of Mood States. ESQ: Environmental Symptoms Questionnaire. NES: Neurobehavioral Evaluation System. SCL-90-R: Symptom Checklist-90-Revised. PSE: Present State Examination. SF-36: Short Form Health Survey-36.

^2^The adverse event (AE) figures listed here are neuropsychiatric AE, where it is possible to elicit that data from the report. n.d.: not determinable.

^3^There were 73 subjects; however 6 of these had used mefloquine at treatment doses. The remaining 67 had used the drug for chemoprophylaxis.

^4^This was a follow-up study that only considered subjects who had submitted adverse event reports to the national drug regulator.
